# The nonlinear correlation between alanine aminotransferase to high-density lipoprotein cholesterol ratio and the risk of diabetes: a historical Japanese cohort study

**DOI:** 10.1186/s12902-023-01382-7

**Published:** 2023-05-29

**Authors:** Changchun Cao, Haofei Hu, Yong Han, Shuting Yuan, Xiaodan Zheng, Xiaohua Zhang, Yibing Zan, Yulong Wang, Yongcheng He

**Affiliations:** 1 Department of Rehabilitation, Shenzhen Dapeng New District Nan’ao People’s Hospital, No. 6, Renmin Road, Dapeng New District, Shenzhen, 518000 Guangdong Province China; 2 Department of Nephrology, Shenzhen Second People’s Hospital, The First Affiliated Hospital of Shenzhen University, Shenzhen, 518000 Guangdong Province China; 3 Department of Emergency, Shenzhen Second People’s Hospital, The First Affiliated Hospital of Shenzhen University, Shenzhen, 518000 Guangdong Province China; 4 Department of Neurology, Shenzhen Samii International Medical Center (The Fourth People’s Hospital of Shenzhen), Shenzhen, 518000 Guangdong Province China; 5 Department of Nephrology, Shenzhen Hengsheng Hospital, Shenzhen, 518000 Guangdong Province China; 6 Department of Nephrology, Affiliated Hospital of North Sichuan Medical College, Nanchong, 637000 Sichuan Province China

**Keywords:** Alanine aminotransferase to high-density lipoprotein cholesterol ratio, Alanine aminotransferase, High-density lipoprotein cholesterol, Diabetes mellitus, Non-linear

## Abstract

**Background:**

Low levels of high-density lipoprotein cholesterol (HDL-C) and high levels of alanine aminotransferase (ALT) are related to insulin resistance, metabolic syndrome, and diabetes mellitus (DM). However, evidence on the connection between the alanine aminotransferase to high-density lipoprotein cholesterol (ALT/HDL-C) ratio and diabetes mellitus (DM) risk was limited. The study aimed to investigate the relationship between baseline ALT/HDL-C ratio and DM among Japanese individuals.

**Methods:**

This second analysis was based on a cohort study using open-source data. Data from 15,342 individuals who participated in the medical examination program were recorded at Murakami Memorial Hospital in Japan between 2004 and 2015. Smooth curve fitting, subgroup analysis, Cox proportional-hazards regression, and a series of sensitivity analyses were conducted to examine the relationship between ALT/HDL-C ratio and incident diabetes. The ability of the ALT/HDL-C ratio to predict diabetes was evaluated using a receiver operating characteristic curve analysis.

**Results:**

After controlling for confounding covariates, the ALT/HDL-C ratio was found to be positively correlated to the DM risk in Japanese adults (HR: 1.01, 95%CI: 1.00–1.02, *P* = 0.049). This study also found a stable relationship between ALT/HDL-C ratio and diabetes after employing a series of sensitivity analyses. Additionally, there was a non-linear association between the ALT/HDL-C ratio and incident diabetes, and the ALT/HDL-C ratio inflection point was 30.12. When the ALT/HDL-C ratio was below 30.12, the present study discovered a significant positive association between the ALT/HDL-C ratio and incident diabetes (HR: 1.04, 95%CI: 1.02–1.06, *P* = 0.001). Furthermore, among liver enzymes, blood lipids, and anthropometric indicators, the ALT/HDL-C ratio best predicts DM (AUC = 0.75, 95%CI: 0.73–0.78).

**Conclusion:**

Increased ALT/HDL-C ratio levels at baseline correlated to incident DM. The relationship between ALT/HDL-C ratio and incident DM was also non-linear. When the ALT/HDL-C ratio is below 30.12, there is a statistically significant positive correlation between the ALT/HDL-C ratio and incident DM.

**Supplementary Information:**

The online version contains supplementary material available at 10.1186/s12902-023-01382-7.

## Introduction

According to a recent estimate from the International Diabetes Federation, there were 463 million individuals with diabetes mellitus (DM) worldwide in 2019, and that number is expected to rise to 578 million by 2030 and 700 million by 2045 [1]. DM is a growing burden on global public health. By 2030, it is predicted that the cost of treating diabetes worldwide will reach at least US$2.1 trillion [2]. Diabetes complications, such as diabetic retinopathy, cardiovascular events, and diabetic nephropathy, significantly negatively impact people's health [3–5]. Strict glucose management can effectively reduce the incidence of these complications [6]. According to earlier clinical trials, individualized intervention successfully lowers or delays the start of DM in a high-risk population [7–9]. Therefore, a new, reliable, and simple test predictor for identifying and treating individuals at high risk for developing diabetes could be beneficial for both cost and health.

Nonalcoholic fatty liver disease (NAFLD) is the most common liver condition in the world, with an estimated 15–40% global prevalence quickly rising [10]. According to reports, people with NAFLD have a twofold higher chance of getting T2DM than people in the general population [11]. High-density lipoprotein cholesterol (HDL-C) and alanine aminotransferase (ALT) were linked to the risk of NAFLD and T2DM, according to some recent research [12–14]. Increased ALT was closely associated with systemic and hepatic insulin resistance (IR) [12]. A decreased concentration of HDL-C was one of the manifestations of metabolic syndrome [15]. Evidence demonstrated impaired HDL-C cholesterol efflux capacity in NAFLD [16]. The antioxidant function of HDL-C may contribute to NAFLD pathogenesis [17]. Previous studies have demonstrated that AST, HDL-C, and NAFLD are associated with insulin resistance [18–20]. Since ALT and HDL-C are linked to DM [13, 20], so we calculated their ratio. We speculated that an elevated alanine aminotransferase to high-density lipoprotein cholesterol (ALT/HDL-C) ratio might be associated with an increased risk of diabetes. Therefore, we undertook cohort research to test this hypothesis to investigate the relationship between the ALT/HDL-C ratio and the risk of diabetes in a sizable Japanese cohort.

## Methods

### Data source

Researchers can freely obtain and access original study data from the Dryad Digital Repository (https://datadryad.org/). Data were obtained from the Dryad data repository for 15,464 participants who did not have diabetes mellitus at baseline (dataset: 10.5061/dryad.8q0p192) [21]. This study used open-source data from the NAGALA database as a secondary investigation of a medical examination program (NAFLD in Gifu Area, Longitudinal Analysis). The previous original study investigated the effect of obesity phenotypes on the risk of incident type 2 diabetes using the NAGALA database [21]. The center where the programs were performed was founded in 1994, evaluated > 8000 medical exams annually, and 60% of participants received one to two exams per year [21]. Since a large percentage of participants underwent repeated examinations, the original study included all participants who underwent repeated examinations between 2004 and 2015 [21]. Researchers are permitted to use the data for secondary analysis under the Dryad terms of service without affecting the authors. The prior study explained the data collection and participant exclusion criteria [21].

### Study participants

The Murakami Memorial Hospital Ethics Committee approved the initial study, and each participant was given written informed consent [21]. The present study used open-source data from the initial study as a secondary investigation. Therefore, this secondary analysis did not need ethical approval. The present study was also carried out under the Declaration of Helsinki. All procedures, including the declarations in the Declarations section, were conducted following the relevant norms and laws.

**Fig. 1:**
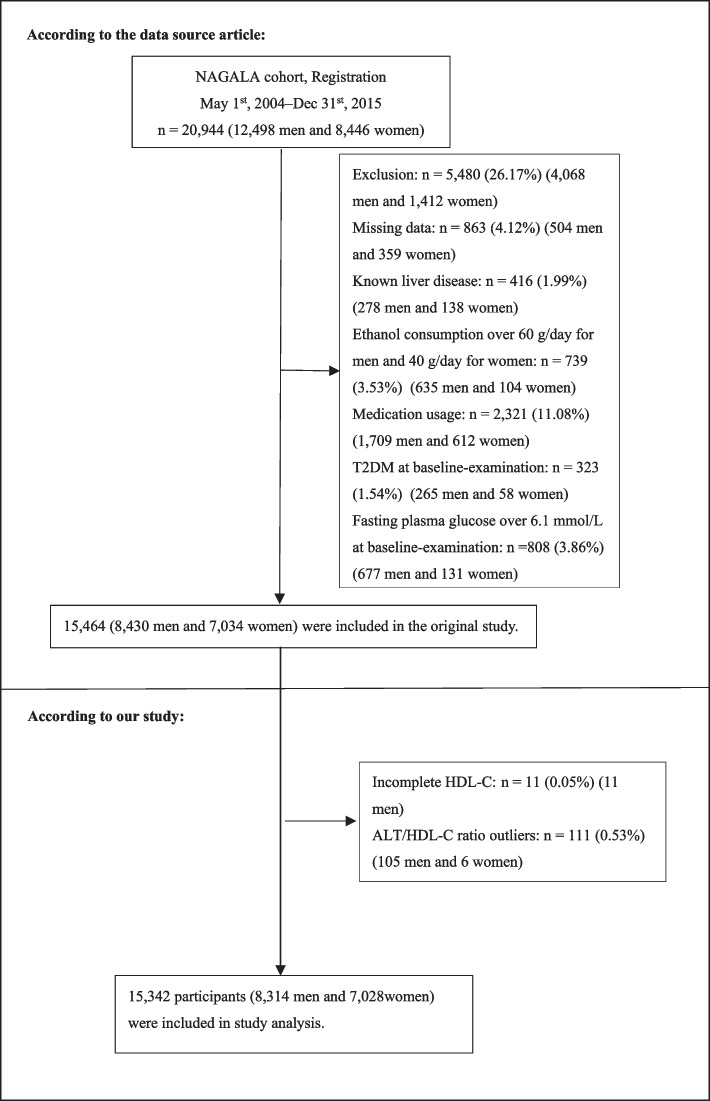
Flowchart of study participants. Figure 1 showed the inclusion of participants. 15,464 participants were assessed for eligibility in the original study. We further excluded 11 participants. The final analysis included 15,342 subjects in the present study

Twenty thousand nine hundred forty-four Japanese people who underwent a physical examination at least twice between 2004 and 2015 were initially included in the study [21]. Afterward, 5602 (26.75%) individuals were eliminated, leaving 15,342 individuals (8314 male and 7028 female) for our study's data analysis (Fig. 1). The following conditions led to the exclusion of individuals from the study: (1) fasting plasma glucose(FPG) ≥ 6.1 mmol/L or type 2 diabetes; (2) known liver disease (such as hepatitis B or hepatitis C at baseline); (3) heavy alcohol consumption (more than 40 g per day for women and 60 g per day for men); (4) taking any medication at baseline; (5) missing data of variables; (6) incomplete HDL-C; (7) those with ALT/HDL-C ratio outliers (three standard deviations above or below three standard deviations from the mean) [22].

### Covariates

Our clinical knowledge and previous research were taken into consideration when selecting the variables for this study. Based on the principles mentioned earlier, the following variables were used as covariates: (1) categorical variables: smoking habits, physical activity, and gender; (2) continuous variables: FPG, ethanol consumption, diastolic blood pressure (DBP), systolic blood pressure (SBP), age, waist circumference (WC), gamma-glutamyl transferase (GGT), aspartate aminotransferase (AST), total cholesterol (TC), glycosylated hemoglobin (HbA1c), triglyceride (TG), and body mass index (BMI). This study's clinical baseline information, including medical history, drinking and smoking habits, and physical activity, was collected using a standardized self-administered questionnaire. The skilled staff carefully measured the subject's height, weight, WC, and blood pressure [21]. Venous blood was collected to measure hematological markers such as TC, TG, ALT, AST, HbA1c, and FPG after the subject had fasted the previous night.

### ALT/HDL-C ratio

Blood samples were collected from the participants after 8 h of fasting. Samples were centrifuged immediately and were stored at -80 °C until analysis. Fasting blood samples were analyzed for HDL-C and ALT [21]. Then, we generated a new variable by dividing ALT to HDL, which was used as the basis for our analysis.

### Diagnosis of incident diabetes

Diabetes was defined as glycosylated hemoglobin ≥ 6.5%, fasting plasma glucose ≥ 7 mmol/L, or self-reported during the follow-up period [23].

### Statistical analysis

Statistical analysis was employed in Empower Stats (R) version 2.2 (www.empowerstats.com, X&Y Solutions, Inc., Boston, MA) and R software version 3.6.1(http://www.R-project.org/).

The ALT/HDL-C ratio was stratified into four groups: Q1 ≤ 7.91; 7.91 < Q2 ≤ 11.52; 11.52 < Q3 ≤ 18.09; Q4 > 18.09. Categorical variables were shown as frequencies and percentages, whereas continuous variables were shown as median and interquartile ranges (25th-75th percentile) or mean ± standard deviation (SD). The Kruskal Wallis H (skew distribution) test, one-way ANOVA (normal distribution), and chi-square test (categorical variable) were employed to analyze differences among different ALT/HDL-C ratio groups.

We describe the incidence rate of diabetes in terms of cumulative and person-year incidence. Kaplan–Meier curves with the log-rank test were employed to demonstrate and investigate the differences in the development of DM during the study period among different ALT/HDL-C ratio groups [24].

After collinearity screening, we used univariate and multivariate Cox proportional-hazards regression models to examine the relationship between the ALT/HDL-C ratio and the risk of developing DM. In addition, we created three models (Model I, Model II, and Model III) to evaluate the connection between the ALT/HDL-C ratio and the risk of developing DM. When these covariances were added to the adjusted model, we would adjusted them if the hazard ratios changed by at least 10% [25]. Additionally, based on the results of the collinearity screening, DBP was excluded from the final multivariate Cox proportional hazards regression equation since it was collinear with other variables (Supplementary Table S1).

The present study used a variety of sensitivity analyses to check whether the conclusions were reliable. In order to confirm the results of the ALT/HDL-C ratio as the continuous variable and check for nonlinearity, we turned the ALT/HDL-C ratio into a categorical variable based on the quartile and calculated the P for the trend. To test the robustness of our results, the present study also used a generalized additive model (GAM) to include the continuous variables as a curve in the equation [26]. In addition, P values were calculated for each pair of groups (total three comparisons: Q1 vs. Q2, Q1 vs. Q3, Q1 vs. Q4), with Bonferroni correction.

Because the ALT/HDL-C ratio was a continuous variable, smooth curve fitting and GAM were conducted to find non-linear correlations. A two-piecewise linear regression model was used to determine the inflection point of the ALT/HDL-C ratio on diabetes risk in terms of the smoothing plot in the event that there was a non-linear connection. The current investigation used the log-likelihood ratio to describe the ideal model for the relationship between the ALT/HDL-C ratio and DM risk.

Additionally, the present study employed the Cox proportional hazard model to analyze the subgroups (BMI, age, habit of exercise, gender, smoking status, hypertension, and ethanol consumption). BMI (< 25, ≥ 25 kg/m^2^), ethanol consumption (< 40, 40–140, 140–280, ≥ 280), hypertension (SBP ≥ 140 mmHg, or DBP ≥ 90 mmHg), and age (< 60, ≥ 60 years) were transformed into categorical variables in accordance with the clinical cut point [27]. And every stratification has undergone a thoroughly adjusted analysis except for the stratification variable. The current study conducted a likelihood ratio test to confirm the interactions between subgroups.

The ability of the ALT/HDL-C ratio, ALT, HDL-C, AST, TC, GGT, TG, WC, and BMI to predict the risk of DM was estimated using the Receiver Operating Characteristic (ROC) curve. For all results, the STROBE statement was followed [28]. Statistical significance was determined by P < 0.05 in two-tailed tests.

## Results

### Characteristics of participants

In the present study, we included a total of 15,342 individuals who were deemed to be free of diabetes at baseline. The average age was 43.70 ± 8.90 years, and 54.19% were male. Our study found that 356 individuals eventually got diabetes after an average of 2206.32 ± 1379.62 days of follow-up. Table 1 displays fundamental indicators, laboratory tests, and other factors. The ALT/HDL-C ratio quartiles (Q1 ≤ 7.91; 7.91 < Q2 ≤ 11.52; 11.52 < Q3 ≤ 18.09; Q4 > 18.09) were used to divide the subjects into four groups. The Q4 groups had higher FPG, WC, ethanol consumption, BMI, GGT, TG, ALT, TC, AST, and low HDL-C than the other three groups. Additionally, there were more men and smokers in the Q4 group.

**Table 1:** The baseline characteristics of Japanese population based on quartiles of ALT/HDL-C ratio

**ALT/HDL-C ratio**	Q1(≤ 7.91)	Q2(7.91 to ≤ 11.52)	Q3(11.52 to ≤ 18.09)	Q4 > 18.09	*P*-value
**Participants**	3836	3830	3837	3839	
**Gender**					< 0.001^a^
Female	3142 (81.91%)	2288 (59.74%)	1220 (31.80%)	378 (9.85%)	
Male	694 (18.09%)	1542 (40.26%)	2617 (68.20%)	3461 (90.15%)	
**Age(years)**	42.03 ± 8.38	43.91 ± 8.97	45.09 ± 9.41	43.94 ± 8.56	< 0.001^b^
**Ethanol consumption(g/week)**	1 (0, 22)	1 (0, 54)	2.8 (0, 84)	12(1, 90)	< 0.001^c^
**Smoking status**					< 0.001^a^
Never-smoker	3004 (78.31%)	2574 (67.21%)	1925 (50.17%)	1485 (38.68%)	
Ex-smoker	428 (11.16%)	600 (15.67%)	876 (22.83%)	1010 (26.31%)	
Current-smoker	404 (10.53%)	656 (17.13%)	1036 (27.00%)	1344 (35.01%)	
**Habit of exercise**					< 0.001^a^
No	3214 (83.79%)	3114 (81.31%)	3056 (79.65%)	3257 (84.84%)	
Yes	622 (16.21%)	716 (18.69%)	781 (20.35%)	582 (15.16%)	
**SBP (mmHg)**	108.82 ± 13.41	111.96 ± 14.45	115.60 ± 14.31	121.25 ± 14.63	< 0.001^b^
**DBP (mmHg)**	67.34 ± 9.54	69.66 ± 9.95	72.57 ± 10.00	76.52 ± 10.12	< 0.001^b^
**BMI (kg/m**^**2**^**)**	20.35 ± 2.26	21.18 ± 2.56	22.39 ± 2.83	24.39 ± 3.03	< 0.001^b^
**WC (cm)**	70.50 ± 6.74	73.54 ± 7.51	77.79 ± 8.05	83.63 ± 7.87	< 0.001^b^
**ALT (IU/L)**	14.47 ± 3.88	16.67 ± 4.30	18.31 ± 5.18	23.05 ± 8.28	< 0.001^b^
**AST (IU/L)**	10.92 ± 2.76	15.02 ± 3.15	19.24 ± 4.21	32.34 ± 12.63	< 0.001^b^
**GGT(IU/L)**	11 (9, 14)	13 (11, 17)	16 (13, 23)	24 (17, 37)	< 0.001^c^
**HDL-C (mmol/L)**	1.82 ± 0.38	1.56 ± 0.32	1.35 ± 0.28	1.13 ± 0.26	< 0.001^b^
**TG (mmol/L)**	0.52 (0.38, 0.70)	0.62 (0.44, 0.87)	0.82 (0.58, 1.16)	1.21 (0.82, 1.74)	< 0.001^c^
**TC (mmol/L)**	5.06 ± 0.82	5.03 ± 0.85	5.13 ± 0.87	5.27 ± 0.88	< 0.001^b^
**HbA1c (%)**	5.15 ± 0.30	5.16 ± 0.32	5.17 ± 0.33	5.21 ± 0.34	< 0.001^b^
**FPG (mmol/L)**	5.00 ± 0.39	5.08 ± 0.41	5.21 ± 0.40	5.35 ± 0.37	< 0.001^b^
**ALT/HDL-C ratio**	6.07 ± 1.26	9.65 ± 1.03	14.36 ± 1.89	29.22 ± 11.44	< 0.001^b^

Values are n(%) or mean ± SD

*ALT/HDL-C ratio* Alanine aminotransferase to high-density lipoprotein cholesterol ratio, *BMI* Body mass index, *WC* Waist circumference, *SBP* Systolic blood pressure, *DBP* Diastolic blood pressure, *ALT* Alanine aminotransferase, *AST* Aspartate aminotransferase, *GGT* Gamma-glutamyl transferase, *HDL-C* High-density lipoprotein cholesterol, *TC* Total cholesterol, *TG* Triglycerides, *HbA1c* Hemoglobin A1c, *FPG* Fasting plasma glucose

^a^represents that the chi-square test was applied

^b^represents that the one-way ANOVA was applied

^c^represents that the Kruskal Wallis H test was applied

### The incidence rate of DM

Table 2 displays the incidence of DM in 356 individuals over the duration of follow-up. All people had an incidence rate of 2.32% (2.08%-2.56%). The four ALT/HDL-C ratio groups' incidence rate were specifically 0.44% (0.23%-0.65%), 1.01% (0.70%-1.34%), 2.19% (1.73%-2.65%), and 5.62% (4.90%-6.36%). In addition, the cumulative incidence rate of the overall population and four ALT/HDL-C ratio groups were 380.72 per 100,000 person-years, 79.18 per 100,000 person-years, 168.18 per 100,000 person-years, 356.87 per 100,000 person-years, and 880.20 per 100,000 person-years, respectively. In contrast to individuals with lower ALT/HDL-C ratio groups, those with higher ALT/HDL-C ratio groups had a higher incidence and cumulative incidence of DM (*P* < 0.001 for trend).

**Table 2:** Incidence rate of incident diabetes based on quartiles of ALT/HDL-C ratio

**ALT/HDL-C ratio**	Participants(n)	DM events(n)	Cumulative incidence (95% CI)(%)	Incidence rate/100000
Total	15,342	356	2.32 (2.08–2.56)	380.72
Q1	3836	17	0.44 (0.23–0.65)	79.18
Q2	3830	39	1.01 (0.70–1.34)	168.18
Q3	3837	84	2.19 (1.73–2.65)	356.87
Q4	3839	216	5.62 (4.90–6.36)	880.20
P for trend			< 0.001	< 0.001

*ALT/HDL-C ratio*, Alanine aminotransferase to high-density lipoprotein cholesterol ratio, *CI* Confidence interval, *DM* Diabetes mellitus

**Fig. 2:**
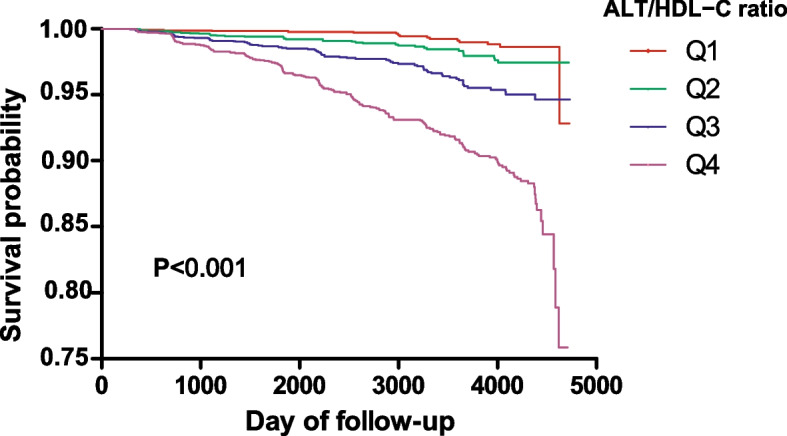
Kaplan–Meier event-free survival curve. Kaplan–Meier event-free survival curve. Kaplan–Meier analysis of incident diabetes based on the ALT/HDL-C ratio quartiles (log-rank, *P* < 0.001)

Figure 2 shows the Kaplan–Meier curves for the likelihood of surviving without DM. Regarding the risk of developing diabetes, there was a significant difference between the four ALT/HDL-C ratio groups (*P* < 0.001). The likelihood of surviving without DM gradually decreased as ALT/HDL-C ratio levels rose. As a result, those in the top ALT/HDL-C ratio groups were most at risk for diabetes.

### Univariate analysis

The results of the univariate analysis were displayed in Table 3 as follows. There was a positive relationship between FPG, DBP, WC, SBP, ethanol consumption, BMI, age, TC, FPG, GGT, TG, HbA1c, ALT, the ALT/HDL-C ratio, AST, and the risk of DM. In contrast, HDL-C is inversely linked to the risk of diabetes. Meanwhile, smokers had a higher risk of diabetes. We also found that men have a higher risk of developing diabetes than women.

**Table 3:** Influencing factors of diabetes using the Univariate Cox proportional-hazards regression model

	**Statistics**	**HR (95% CI)**	***P*** ** value**
**Gender**			< 0.001
Female	7028 (45.81%)	ref	
Male	8314 (54.19%)	2.40 (1.89, 3.06)	
**Age(years)**	43.74 ± 8.90	1.06 (1.05, 1.07)	< 0.001
**Ethanol consumption(g/week)**	47.79 ± 82.34	1.00 (1.00, 1.00)	0.001
**Smoking status**			
Never-smoker	8988 (58.58%)	ref	
Ex-smoker	2914 (18.99%)	1.61 (1.21, 2.14)	0.001
Current-smoker	3440 (22.42%)	2.52 (2.00, 3.18)	< 0.001
**Habit of exercise**			0.126
No	12,641 (82.39%)	ref	
Yes	2701 (17.61%)	0.79 (0.59, 1.07)	
**SBP (mmHg)**	114.41 ± 14.94	1.03 (1.03, 1.04)	< 0.001
**DBP (mmHg)**	71.52 ± 10.48	1.05 (1.04, 1.06)	< 0.001
**BMI (kg/m**^**2**^**)**	22.08 ± 3.09	1.24 (1.22, 1.27)	< 0.001
**WC (cm)**	76.37 ± 9.03	1.10 (1.08, 1.11)	< 0.001
**ALT (IU/L)**	19.38 ± 10.65	1.04 (1.04, 1.05)	< 0.001
**AST (IU/L)**	18.13 ± 6.50	1.04 (1.04, 1.05)	< 0.001
**GGT (IU/L)**	20.07 ± 17.67	1.01 (1.01, 1.01)	
**HDL-C (mmol/L)**	1.47 ± 0.40	0.14 (0.10, 0.20)	< 0.001
**TG (mmol/L)**	0.91 ± 0.65	1.79 (1.67, 1.92)	< 0.001
**TC (mmol/L)**	5.12 ± 0.86	1.48 (1.33, 1.66)	< 0.001
**HbA1c (%)**	5.17 ± 0.32	54.10 (39.06, 74.92)	< 0.001
**FPG (mmol/L)**	5.16 ± 0.41	25.49 (18.66, 34.82)	< 0.001
**ALT/HDL-C ratio**	14.83 ± 10.58	1.05 (1.04, 1.05)	< 0.001

Values are n(%) or mean ± SD

*ALT/HDL-C ratio* Alanine aminotransferase to high-density lipoprotein cholesterol ratio, *BMI* Body mass index, *WC* Waist circumference, *SBP* Systolic blood pressure, *DBP* Diastolic blood pressure, *ALT* Alanine aminotransferase, *AST* Aspartate aminotransferase, *GGT* Gamma-glutamyl transferase, *HDL-C* High-density lipoprotein cholesterol, *TC* Total cholesterol, *TG* Triglycerides, *HbA1c* Hemoglobin A1c, *FPG* Fasting plasma glucose, *HR* Hazard ratio, *CI* Confidence interval, *Ref* Reference

### The connection between the ALT/HDL-C ratio and the incidence of DM

As ALT/HDL-C ratio met the proportional hazards assumption, the association between the ALT/HDL-C ratio and the incident DM was evaluated by the Cox proportional hazards regression model. Table 4 displays the Cox proportional hazard regression models that showed the hazard ratios (HR) and 95% confidence interval (CI) for the connection between the ALT/HDL-C ratio and diabetes. The HR (95% CI) for diabetes connection with the ALT/HDL-C ratio was 1.05 (1.04–1.05) in Model I. After adjusting for gender, ethanol consumption, BMI, habit of exercise, smoking status, SBP and age, the HR (95% CI) was 1.03 (1.02–1.04) in Model II. In Model III, after adjusting for gender, ethanol consumption, BMI, habit of exercise, smoking status, SBP, age, FPG, AST, GGT, HbA1c, TC and TG, the ALT/HDL-C ratio showed an independent association with incident DM, with the HR of 1.11 (1.00, 1.24) per SD (SD = 10.58) increase. The results showed that for every 1 SD rise in the ALT/HDL-C ratio, the risk of diabetes increases by 11%.

**Table 4:** Relationship between ALT/HDL-C ratio and the incident diabetes in different models

Variable	Model I (HR.,95% CI, *P*)	Model II (HR,95% CI, P)	Model III (HR,95% CI, P)	GAM (HR,95% CI, P)
ALT/HDL-C ratio	1.05 (1.04, 1.05) < 0.001	1.03 (1.02, 1.04) < 0.001	1.01 (1.00, 1.02) 0.049	1.01 (1.00, 1.02) 0.0318
ALT/HDL-C ratio (per SD)	1.65 (1.55, 1.74) < 0.001	1.38 (1.28, 1.50) < 0.001	1.11 (1.00, 1.24) 0.049	1.12 (1.00, 1.25) 0.0318
ALT/HDL-C ratio (quartile)				
Q1	ref	ref	ref	ref
Q2	2.11 (1.19, 3.73) 0.031*	1.66 (0.94, 2.95) 0.249*	1.27 (0.71, 2.26) 0.999*	1.27 (0.70, 2.31) 0.999*
Q3	4.44 (2.63, 7.47) < 0.001*	2.65 (1.54, 4.58) 0.001*	1.81 (1.04, 3.14) 0.105*	1.87 (1.04, 3.35) 0.110*
Q4	10.68 (6.52, 17.50) < 0.001*	4.69 (2.70, 8.13) < 0.001*	2.24 (1.25, 4.02) 0.021*	2.52 (1.34, 4.74) 0.013*
P for trend	< 0.001	< 0.001	0.001	0.001

Model I: we did not adjust for other covariants

Model II: we adjusted for gender, age, ethanol consumption, smoking status, habit of exercise, BMI, and SBP

Model III: we adjusted for gender, age, ethanol consumption, smoking status, habit of exercise, BMI, SBP, AST, GGT, TC, TG, HbA1c, and FPG

GAM: we adjusted for gender, age, ethanol consumption, smoking status, habit of exercise, BMI, SBP, AST, GGT, TC, TG, HbA1c, and FPG. However, continuous covariates were adjusted as nonlinearity

*HR* Hazard ratio, *CI* Confidence interval, *Ref* Reference, *SD* Standard deviation, *ALT/HDL-C ratio* Alanine aminotransferase to high-density lipoprotein cholesterol ratio

^*^represents that the Bonferroni correction for multiple comparisons was applied

### Sensitive analysis

We employed several sensitivity analyses to evaluate how reliable our results were. We processed the ALT/HDL-C ratio from a continuous variable to a categorical variable and then reintroduced the categorically transformed ALT/HDL-C ratio into the model. The trend p after handling the ALT/HDL-C ratio as categorical variables were not equal, implying a potential non-linear connection between the ALT/HDL-C ratio and diabetes risk. Moreover, a GAM added the continuity covariate to the equation. We discovered that the GAM model's results were in line with the fully adjusted model (HR: 1.01, 95%CI: 1.00–1.02) (Table 4).

### The analyses of the non-linear relationship

**Fig. 3:**
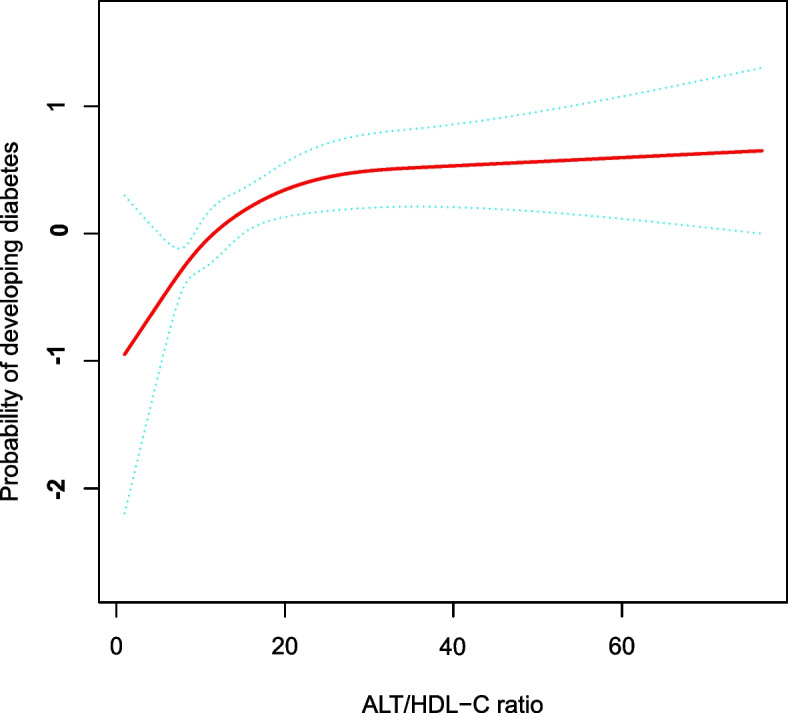
The nonlinear relationship between the ALT/HDL-C ratio and incident diabetes. A nonlinear relationship was detected after adjusting for gender, age, ethanol consumption, smoking status, habit of exercise, BMI, SBP, AST, GGT, TC, TG, HbA1c, and FPG. The light green dot represents 95% CI

Figure 3 displays the smooth curve fitting and GAM that were employed to assess the non-linear correlation between the ALT/HDL-C ratio and diabetes risk. After adjusting for confounding covariates, there was a nonlinear correlation between the ALT/HDL-C ratio and DM (Table 5). The present investigation discovered that the inflection point of the ALT/HDL-C ratio based on a two-piecewise linear regression model was 30.12 (P for log-likelihood ratio test = 0.005). The probability of diabetes was positively correlated with the ALT/HDL-C ratio when the ALT/HDL-C ratio was below 30.12 (HR: 1.04, 95%CI: 1.02–1.06, *P* = 0.001). In contrast, When the ALT/HDL-C ratio was above 30.12, on the other hand, their association was not significant (HR: 1.00, 95%CI: 0.98–1.01, *P* = 0.736).

**Table 5:** The result of the two-piecewise linear regression on the relationship between the ALT/HDL-C ratio and diabetes

Incident DM	HR (95%CI),	*P*
Fitting model by standard linear regression	1.01 (1.00, 1.02)	0.049
Fitting model by two-piecewise linear regression		
Inflection point of ALT/HDL-C ratio	30.12	
≤ 30.12	1.04 (1.02, 1.06)	0.001
> 30.12	1.00 (0.98, 1.01)	0.736
P for the log-likelihood ratio test	0.005	

We adjusted for gender, age, ethanol consumption, smoking status, habit of exercise, BMI, SBP, AST, GGT, TC, TG, HbA1c, and FPG

*HR* Hazard ratios, *CI* Confidence, *DM* Diabetes mellitus, *ALT/HDL-C ratio* Alanine aminotransferase to high-density lipoprotein cholesterol ratio

### The results of the subgroup analysis

Subgroup analysis was employed to investigate other variables that may affect the correlation between the ALT/HDL-C ratio and the incidence of DM. BMI, age, habit of exercise, gender, smoking status, hypertension, and ethanol consumption were selected as stratification variables, and the trends in the effect sizes of these influencing variables were evaluated (Table 6). Smoking status, habit of exercise, ethanol consumption BMI, and gender had no impact on the correlation between the ALT/HDL-C ratio and the incidence of diabetes. And a stronger correlation was discovered in the individuals with age ≥ 60 years, and hypertension. In contrast, there was a weaker correlation in individuals without age ≥ 60 years, and hypertension.

**Table 6:** Effect size of ALT/HDL-C ratio on diabetes in prespecified and exploratory subgroups

Independent variables	No of patients	Effect size(95%CI)	*P* value	*P* for interaction
**Age(years)**				0.015
< 60	14,631	1.01 (1.00, 1.02)	0.300	
≥ 60	711	1.05 (1.02, 1.09)	0.003	
**Gender**				0.158
Female	7028	1.03 (1.00, 1.05)	0.021	
Male	8314	1.01 (1.00, 1.02)	0.069	
**Ethanol consumptio (g/week)**				0.109
< 40	10,685	1.00 (0.99, 1.02)	0.599	
≥ 40, < 140	2769	1.03 (1.01, 1.05)	0.009	
≥ 140, < 280	1352	1.04 (1.00, 1.07)	0.062	
≥ 280	536	1.03 (0.99, 1.07)	0.176	
**Smoking status**				0.336
Never-smoker	8988	1.00 (0.98, 1.02)	0.791	
Ex-smoker	2914	1.02 (1.00, 1.04)	0.042	
Current-smoker	3440	1.02 (1.00, 1.03)	0.0370	
**Habit of exercise**				0.055
No	12,641	1.01 (1.00, 1.02)	0.008	
Yes	2701	0.98 (0.95, 1.01)	0.2700	
**Hypertension**				0.040
No	14,399	1.01 (0.99, 1.02)	0.357	
Yes	943	1.04 (1.01, 1.06)	0.009	
**BMI (kg/m**^**2**^**)**				0.486
< 25	12,905	1.02 (1.00, 1.03)	0.023	
≥ 25	2437	1.01 (0.99, 1.02)	0.233	

Note 1: Above model adjusted for we adjusted for gender, age, ethanol consumption, smoking status, habit of exercise, BMI, SBP, AST, GGT, TC, TG, HbA1c, and FPG

Note 2: The model is not adjusted for the stratification variable in each case

### DM prediction using the ALT/HDL-C ratio

**Fig. 4:**
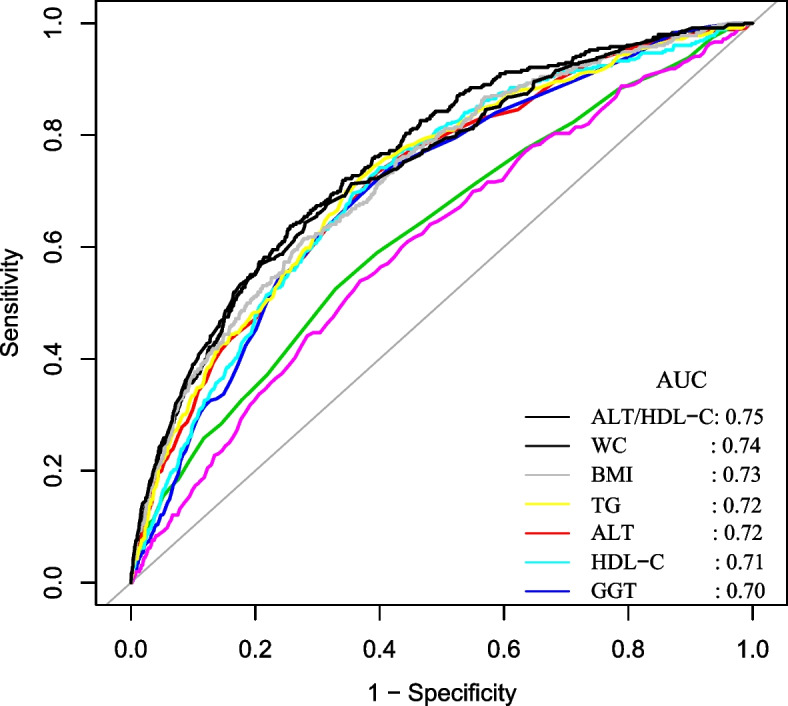
The ALT/HDL-C ratio for predicting DM in all participants by ROC analyses. Among liver enzymes, blood lipids, and anthropometric indicators, including ALT, GGT, AST, TC, TG, HDL-C, BMI, and WC, the AUC of the ALT/HDL-C ratio for predicting DM was the highest

The AUC of the ALT/HDL-C ratio was 0.75 for predicting DM (Table 7 and Fig. 4). Among liver enzymes, blood lipids, and anthropometric indicators, including ALT, GGT, AST, TC, TG, HDL-C, BMI, and WC, the AUC of the ALT/HDL-C ratio for predicting DM was the highest, as shown in Fig. 4. Therefore, the ALT/HDL-C ratio can be used as a secondary marker to predict diabetes compared to ALT, GGT, AST, TC, TG, HDL-C, BMI and WC.

**Table 7:** Areas under the receiver operating characteristic curves (AUROC) for each evaluated parameter in identifying diabetes

Test	AUROC	95%CI	Best threshold	Specificity	Sensitivity	Youden Index
ALT/HDL-C ratio	0.75	0.73–0.78	17.56	0.74	0.64	0.38
ALT	0.72	0.69–0.74	18.5	0.6	0.73	0.34
AST	0.63	0.60–0.66	19.5	0.67	0.53	0.2
GGT	0.7	0.68–0.73	16.5	0.59	0.74	0.33
HDL-C	0.71	0.69–0.74	1.33	0.6	0.74	0.34
TC	0.6	0.57–0.63	5.37	0.63	0.54	0.17
TG	0.72	0.70–0.75	0.89	0.63	0.72	0.35
BMI	0.73	0.70–0.75	23.53	0.72	0.62	0.34
WC	0.74	0.71–0.77	81.05	0.72	0.64	0.36

## Discussion

Our historical study using open-source data investigated the connection between the ALT/HDL-C ratio and the risk of developing DM in Japanese participants. The present study showed that a higher ALT/HDL-C ratio was linked to a higher risk of DM. The correlation between ALT/HDL-C ratio and diabetes was also examined on the right and left sides of the inflection point. The ALT/HDL-C ratio has a non-linear correlation with the incidence of DM. It was found that individuals with age ≥ 60 years, and hypertension had a greater correlation between the ALT/HDL-C ratio and the incidence of DM. Furthermore, among the other indices, such as GGT, TG, AST, TC, and WC, the ALT/HDL-C ratio had the highest AUC for predicting DM.

The age-standardized incidence rate of diabetes was 8.8/1000 person-years in Japan during the same period. The age-standardized incidence rate of diabetes in the present study was 3.80 per 1000 person-years, lower than the reported level during the same period [29]. This apparent difference may be due to the more detailed data analysis and more stringent selection criteria used in this study, such as the exclusion of heavy drinking habits, viral hepatitis, and FPG ≥ 6.1 mmol/L.

Recent evidence has revealed that increased ALT is an independent risk factor for the development of DM. A historical study demonstrated that compared to the Q1 group of ALT, the HR for DM in the Q4 group was 1.89 (95%CI: 1.26–2.83) [12]. Another study suggested that high ALT is strongly and independently related to a higher DM risk (HR:1.23, 95%CI: 1.10–1.50) after adjusting for consumption of alcohol, history of diabetes, age, degree of education, and smoking history [14]. In addition, much evidence suggested that elevated HDL-C was independently and strongly related to reduced risk of diabetes after adjusting for confounding factors [13]. Although studies on the relationship between ALT/HDL-C ratio and DM risk are lacking, an elevated ALT/HDL-C ratio indicates elevated ALT or decreased HDL-C. As a result, our findings agreed with the earlier findings. It should be noted that, compared with the study of Zhang J et al. [14], we adjusted for confounding factors such as AST, FPG, TG, and HbA1c. Recent research has, however, identified these variables as diabetes-related risk factors.

Furthermore, the current study first examined the nonlinear connection between the ALT/HDL-C ratio and DM. After controlling for gender, ethanol consumption, BMI, habit of exercise, smoking status, SBP, age, FPG, AST, GGT, HbA1c, TC and TG, the smooth curve result revealed that the connection between the ALT/HDL-C ratio and diabetes was non-linear. The present study used a two-piecewise linear regression model to determine the ALT/HDL-C ratio inflection point. A 1 unit increase in the ALT/HDL-C ratio level was linked to a 4% increase in HR for diabetes when the ALT/HDL-C ratio was less than 30.12 (HR: 1.04, 95%CI: 1.02–1.06, *P* = 0.001). However, the ALT/HDL-C ratio level did not correlate with incident diabetes when the ALT/HDL-C ratio level was above 30.12 (HR: 1.00, 95%CI: 0.98–1.01, *P* = 0.736). Elevated ALT/HDL-C ratio informs the participants at high risk of developing diabetes during follow-up, which will serve as a reminder to change lifestyle habits sooner to improve outcomes. Our findings provide a theoretical basis for reducing the risk of DM in the clinical setting by lowering the ALT/HDL-C ratio, especially when the ALT/HDL-C ratio decreases below the inflection point. It includes additional information to prevent DM in patients with different ALT/HDL-C ratio levels. In addition, the results of this study may be useful in the future to combine with other markers to establish a predictive model of diabetes risk.

The mechanism behind the connection between the ALT/HDL-C ratio and DM is unknown, but IR may be involved. Regarding ALT levels, ALT was connected to hepatic insulin sensitivity as a measure of liver fat formation [30]. ALT is frequently regarded as an epidemiologic indicator of non-alcoholic fatty liver disease linked to a higher risk of acquiring diabetes [31]. Additionally, it has been shown that ALT levels are associated with hepatic insulin resistance, which may aid in the onset of diabetes [18]. Reduced HDL-C levels may negatively affect β cells' ability, decreasing insulin sensitivity and output [32, 33]. Therefore, we propose that by influencing insulin resistance, the ALT/HDL-C ratio may influence the onset of DM. By measuring the degree of insulin resistance, we can eventually confirm the precise mechanism of action between the ALT/HDL-C ratio and DM.

Our study has several following advantages. First, we explored the non-linear connection between the ALT/HDL-C ratio and DM. Second, residual confounding factors were minimized by using strict statistical adjustments. Third, sensitivity analyses were conducted to ensure the robustness of the results. It included transforming the ALT/HDL-C ratio into a categorical variable and using a GAM to insert the continuity covariate into the equation as a curve. Fourth, the present study conducted a group analysis to assess other risk factors that might influence the connection between the ALT/HDL-C ratio and diabetes.

The present study does have certain restrictions. First, diabetes may have been underestimated due to a lack of experimental OGTT. Second, because the present study is a secondary analysis, it is not possible to make adjustments for factors like insulin resistance, renal function, heavy alcohol consumption, and liver diseases that were not present in the initial dataset. In the future, we can consider designing our studies or collaborating with other researchers to collect as many variables as possible, including information regarding heavy alcohol consumption and liver diseases. After adjusting for insulin resistance, renal function, heavy alcohol consumption, and liver diseases, we will analyze the relationship between ALT/HDL ratio and diabetes. Third, The original study did not collect some covariates such as smoking, age, blood pressure, and laboratory data that were time-varying variables. Fourth, the initial study didn't cover how ALT and HDL-C fluctuated over time. Future designs of our investigation may include capturing additional variables, such as variations in ALT and HDL-C during follow-up. As a result, we might use a GAM model to investigate how changes in the ALT/HDL-C ratio would affect future diabetes risk. Fifth, our study had a high rate of loss to follow-up. Among the 20,944 people, 5602 (27%) were removed from the study. Since this study is based on a secondary analysis of public data, the original study did not provide information on missing persons. So we do not have access to missing person information. In the future, we will conduct our research study. We can examine the characteristics of those lost to follow-up and compare them to those who remain in the study. Sixth, the HR between the ALT/HDL-C ratio and diabetes was small, and the AUC of the ALT/HDL-C ratio was 0.75 for predicting DM. Therefore, in clinical practice, the ALT/HDL ratio is difficult to use primarily as a predictor of diabetes. In the future, we will conduct our study to evaluate the combination of ALT/HDL-C ratio and other markers for predicting diabetes risk.

## Conclusion

This study shows that the Japanese population's ALT/HDL-C ratio and incident DM have a positive and non-linear connection. The relationship between the ALT/HDL-C ratio level and incident DM had a threshold impact. When the ALT/HDL-C ratio is below 30.12, there is a statistically significant positive correlation between the ALT/HDL-C ratio and incident DM. The outcomes were anticipated to serve as a guide for clinicians managing the ALT/HDL-C ratio. According to this study, the ALT/HDL-C ratio might be used as a secondary marker to predict diabetes.

## Supplementary Information

**Additional file 1: Table S1. **Collinearity screening. **Table S2. **Relationship between ALT/HDL-C ratio and the incident diabetes. **Table S3.** Relationship between ALT/HDL-C ratio and the incident diabete using Negative Binomial Regression. **Table S4.** Relationship between the ALT/HDL-C ratio and incident diabetes in different sensitivity analyses.

## Data Availability

The raw data can be downloaded from the ‘DATADRYAD’ database (www.Datadryad.org). Dryad Digital Repository. https://datadryad.org/stash/dataset/doi:10.5061%2Fdryad.8q0p192.
