# Supplementary material for: The nonlinear correlation between alanine aminotransferase to high-density lipoprotein cholesterol ratio and the risk of diabetes: a historical Japanese cohort study
Source: BMC Endocr Disord. 2023 May 29;23:124. doi: 10.1186/s12902-023-01382-7 (PMC10226242; doi:10.1186/s12902-023-01382-7)
Supplement: Supplementary file 1 — Additional file 1: Table S1. Collinearity screening. Table S2. Relationship between ALT/HDL-C ratio and the incident diabetes. Table S3. Relationship between ALT/HDL-C ratio and the incident diabete using Negative Binomial Regression. Table S4. Relationship between the ALT/HDL-C ratio and incident diabetes in different sensitivity analyses. [file 12902_2023_1382_MOESM1_ESM.docx]

**Table S1** collinearity screening

|  | Step 1 | Step 2 |
| --- | --- | --- |
| Gender | 1.8 | 1.8 |
| Age (years) | 1.2 | 1.2 |
| Ethanol consumption(g/week) | 1.3 | 1.3 |
| Smoking status | 1.4 | 1.4 |
| Habit of exercise | 1 | 1 |
| SBP (mmHg) | 5.6 | 1.4 |
| DBP (mmHg) | 5.7 | NA |
| BMI (kg/m2) | 1.5 | 1.5 |
| AST (IU/L) | 1.2 | 1.2 |
| GGT(IU/L) | 1.4 | 1.4 |
| TG (mmol/L) | 1.5 | 1.5 |
| TC (mmol/L) | 1.2 | 1.2 |
| HbA1c (%) | 1.2 | 1.2 |
| FPG (mmol/L) | 1.5 | 1.5 |

NA was the excluded variable

BMI: body mass index; SBP: systolic blood pressure; DBP: diastolic blood pressure; ALT: alanine aminotransferase; AST: aspartate aminotransferase; GGT: gamma-glutamyl transferase; HDL-C: high-density lipoprotein cholesterol; TC: total cholesterol; TG: triglycerides; HbA1c: hemoglobin A1c; FPG: fasting plasma glucose.

**Table S2** Relationship between ALT/HDL-C ratio and the incident diabetes

| Variable | Model I (HR.,95% CI, P) | Model II (HR,95% CI, P) | Model III (HR,95% CI, P) |
| --- | --- | --- | --- |
| ALT/HDL-C ratio | 1.052 (1.050, 1.053) <0.0001 | 1.032 (1.029, 1.035) <0.0001 | 1.015 (1.011, 1.019) <0.0001 |
| ALT/HDL-C ratio (quartile) |  |  |  |
| Q1 | ref | ref | ref |
| Q2 | 2.294 (1.970, 2.671) <0.0001 | 1.583 (1.272, 1.971) <0.0001 | 1.332 (1.068, 1.660) 0.0109 |
| Q3 | 4.936 (4.294, 5.674) <0.0001 | 2.865 (2.331, 3.522) <0.0001 | 2.059 (1.671, 2.538) <0.0001 |
| Q4 | 12.686 (11.118, 14.475) <0.0001 | 5.732 (4.650, 7.067) <0.0001 | 3.159 (2.530, 3.946) <0.0001 |
| P for trend | <0.0001 | <0.0001 | <0.0001 |

Model I: we did not adjust for other covariants.

Model II: we adjusted for gender, change of age, ethanol consumption, smoking status, habit of exercise, BMI, and SBP.

Model III: we adjusted for gender, change of age, ethanol consumption, smoking status, habit of exercise, BMI, SBP, AST, GGT, TC, TG, HbA1c, and FPG.

**Table S3** Relationship between ALT/HDL-C ratio and the incident diabete using Negative Binomial Regression

|  | β | Std. Error | HR | 95%CI low | 95%CI upp | P |
| --- | --- | --- | --- | --- | --- | --- |
| ALT/HDL-C ratio | 0.03313 | 0.00609 | 1.03369 | 1.02143 | 1.04609 | <0.000001 |

Note: we adjusted for gender, age, ethanol consumption, smoking status, habit of exercise, BMI, SBP, AST, GGT, TC, TG, HbA1c, and FPG.

**Table S4** Relationship between the ALT/HDL-C ratio and incident diabetes in different sensitivity analyses

| Exposure | Model I (HR,95%CI, P) | Model II (HR,95%CI, P) |
| --- | --- | --- |
| **ALT/HDL-C ratio** | 1.01 (1.00, 1.03) 0.046 | 1.01 (1.00, 1.02) 0.049 |
| **ALT/HDL-C ratio (quartile)** |  |  |
| Q1 | ref | ref |
| Q2 | 1.34 (0.73, 2.47) 0.999* | 1.37 (0.75, 2.48) 0.916* |
| Q3 | 1.62 (0.89, 2.97) 0.351* | 1.80 (1.02, 3.19) 0.126* |
| Q4 | 2.15 (1.11, 4.18) 0.072* | 2.24 (1.22, 4.08) 0.026* |
| P for trend | 0.015 | 1.29 (1.09, 1.53) 0.003 |

Model, I was sensitivity analysis after excluding those with BMI≥25kg/m^2^. We adjusted gender, age, ethanol consumption, smoking status, habit of exercise, BMI, SBP, AST, GGT, TC, TG, HbA1c, and FPG..

Model II was sensitivity analysis after excluding those with ethanol consumption ≥ 280g/week. We adjusted gender, age, ethanol consumption, smoking status, habit of exercise, BMI, SBP, AST, GGT, TC, TG, HbA1c, and FPG..

HR: hazard ratios; CI: confidence; Ref: reference; ALT/HDL-C ratio: alanine aminotransferase to high-density lipoprotein cholesterol ratio.

*represents that the Bonferroni correction for multiple comparisons was applied.
